# Profiles of Physical Fitness Risk Behaviours in School Adolescents from the ASSO Project: A Latent Class Analysis

**DOI:** 10.3390/ijerph15091933

**Published:** 2018-09-05

**Authors:** Garden Tabacchi, Avery Faigenbaum, Monèm Jemni, Ewan Thomas, Laura Capranica, Antonio Palma, Joao Breda, Antonino Bianco

**Affiliations:** 1 Sport and Exercise Sciences Unit, SPPF Department, University of Palermo, Via Giovanni Pascoli 6, 90144 Palermo, Italy; tabacchi.garden@libero.it (G.T.); antonio.palma@unipa.it (A.P.); antonino.bianco@unipa.it (A.B.); 2 Department of Health and Exercise Science, The College of New Jersey, 2000 Pennington Rd Ewing, NJ 08628, USA; faigenba@tcnj.edu; 3 ISAFA—International Science and Football Association, 13 Musker Pl, Papworth Everard, Cambridge CB23 3LE, UK; monemj@hotmail.com; 4 Department of Movement, Human and Health Sciences, University of Rome Foro Italico, P.za Lauro de Bosis 15, 00135 Rome, Italy; laura.capranica@uniroma4.it; 5 Division of Non-communicable Diseases and Life-Course, World Health Organization Regional Office for Europe, UN City, Marmorvej 51, DK, 2100 Copenhagen, Denmark; rodriguesdasilvabred@who.int

**Keywords:** latent class analysis, physical fitness, health, adolescents

## Abstract

The aim of the present investigation was to describe profiles of adolescents’ fitness level, identify latent classes of fitness-related risk behaviours, and describe their sociodemographic and environmental predictors. In total, 883 adolescents (16.4 ± 1.4 years; 167.3 ± 10.4 cm; 62.8 ± 13.5 kg; 62.2% males) were assessed for personal and lifestyle information and for physical fitness components. Eleven possible fitness determinants and seven predictors were included. Latent class analysis (LCA) was used to determine fitness-related risk behaviours. Logistic regressions predicted class membership and assessed associations with fitness levels and fitness components. Five latent classes were recognised: 1—virtuous, 30.7% of respondents; 2—low physical activity/sport, 18.8%; 3—incorrect alcohol/food habits, 25.8%; 4—health risk/overweight, 15.9%; 5—malaise/diseases, 8.8%. Sex, age, parents’ overweightness/obesity and education, and school type predicted most classes significantly. Compared to class 1, class 2 had higher odds of having all poor fitness components except upper body maximal strength; class 4 had higher risk of low muscular endurance; and class 5 was likely to have lower maximal strength, muscular endurance, and speed/agility. Educating adolescents to reach a sufficient practice of PA/sport could help decreasing the risk of low health-related fitness more than discouraging them from using alcohol, addressing proper food behaviours and habits, and helping them understand their psychophysical malaise symptoms.

## 1. Introduction

Low levels of health-related physical fitness (HRF) in youth can influence mortality and morbidities in adulthood for several disorders, including cardiovascular disease, obesity, and metabolic syndrome [1,2,3,4,5]. It has been reported that fitness is a better predictor of health outcomes in adults than physical activity (PA) levels [6]. In children, data from cross-sectional and prospective studies have been used to suggest that increasing PA is insufficient since future cardiovascular risk is more dependent on fitness than on the amount of PA performed [7,8]. In particular, inadequate levels of aerobic fitness in children and adolescents can influence overweightness, metabolic disorders, and cognitive diseases that predict morbidities in adulthood [9,10,11,12]. Thus, it is important to establish healthy habits early in life to attain a desirable level of fitness during childhood and adolescence [13] by acting on the factors that could influence this behaviour [14]. As noted in other reports, adolescents’ low fitness levels could be due to several factors involving genetic, biological, familial, environmental, and behavioural aspects [13,15,16]. At present, female sex, low income, low consumption of dairy products and bread/cereals, increased consumption of sweetened beverages, insufficient PA level, excessive screen time, and excess body fat have been found to be associated with low aerobic fitness levels in youth [17,18,19,20]. Additionally, the contemporary construct of paediatric dynapenia, an acquired condition characterized by low levels of muscular strength and power, has been used to identify youth with consequent functional limitations not caused by neurologic or muscular disease [21].

The latent class analysis (LCA) is a promising statistical tool that has been frequently used in recent years to identify small homogenous groups based on behavioural patterns related to health, obesity, diet, PA, sedentariness, substance use, and smoking [22,23,24,25,26,27]. Moreover, LCA allows the identification of latent classes that can be used to target proper interventions [28,29,30]. Of interest, previous investigations have used cluster analysis to identify potential patterns of health-related behaviours in adolescents [31,32,33,34,35], and other colleagues have already used LCA to determine patterns of behaviours related to obesity or to PA and sedentariness on different population targets [26,27,36,37,38,39,40,41]. However, to our knowledge, no studies have identified distinctive classes of patterns with respect to low fitness risk factors. The aims of this paper were to describe profiles of adolescents’ fitness level, identify latent classes of fitness-related risk behaviours, describe sociodemographic and environmental predictors of class membership, and evaluate the association of the identified patterns with overall fitness level and single health-related fitness components.

## 2. Materials and Methods

### 2.1. Study Design and Sample

The ASSO (Adolescents Surveillance System for the Obesity prevention) Project funded by the Italian Ministry of Health was aimed at developing a surveillance system in schools to collect data on adolescents’ lifestyles and food consumption, and anthropometric and fitness measurements [42,43,44].

A total of 883 students were recruited, through a multistage sampling, during the years 2012 and 2013 from the classes 1 to 4 of seven public and private high schools within Palermo city (Italy). All participants were provided with information sheets and had to supply the informed consent signed by their parents before starting the study.

A standardized methodology with standard operating procedures (SOPs) has been developed for the data collection, and training sessions were organized for all the ASSO team members and teachers of the participating schools.

The principles of the Italian data protection (196/2003) were guaranteed and the Ethical Committee of the Azienda Ospedaliera Universitaria Policlinico “Paolo Giaccone” in Palermo approved the project and the study protocol (approval code n.9/2011).

Fitness measures were collected through the ASSO-Fitness Tests Battery (ASSO-FTB) [45,46], composed by five accurately selected tests for the assessment of five physical fitness components: (1) the hand-grip strength test (HG) to assess upper body maximal strength; (2) the standing broad jump test (SBJ) to assess lower body strength and power; (3) the sit-up test to exhaustion (SUe) to assess local muscular endurance; (4) the 4 × 10 m shuttle run test (4 × 10 m SR) to assess speed and agility; and (5) the 20 m shuttle run test (20m SR) to assess endurance/aerobic capacity. Classes of fitness levels were derived, and the detailed description of the applied methodology can be found in Bianco et al. [45]. Fitness measures were complete for all five components for a total of 544 students aged 13–19, with mean age 16.3 ± 1.4 years; M = 369 (67.8%); F = 175 (32.2%). Anthropometric measurements of weight, height, and waist circumference were collected by the teachers through the use of a calibrated scale, a stadiometer, and a nonelastic meter, respectively, all available within the schools. Personal information and lifestyle aspects were collected through the web-based questionnaires of the ASSO-NutFit software administered within the schools: ASSO-PIQ (Personal Information Questionnaire), ASSO-PASAQ (Physical Activity, Smoke, and Alcohol Questionnaire) and ASSO-FHQ (Food Habits Questionnaire). The ASSO-PIQ included questions regarding participant and family information and neonatal and clinical assessment. The ASSO-PASAQ consisted of three sections: physical activity, smoking, and alcoholic drinks and other beverages. Finally, the ASSO-FHQ consisted of six items, regarding: breakfast, school break, lunch, afternoon break, dinner, and various habits such as eating out, eating ready meals, organic food, fresh food, or food from vending machines [42,44,47,48,49].

### 2.2. Variables

Fitness levels and the five fitness components considered (upper body maximal strength, lower body maximal strength, muscular endurance, speed and agility, and endurance/aerobic capacity) were categorized into three classes (0 = high, 1 = medium, and 2 = low), and subsequently for the purpose of the logistic regression analysis into two classes (0 = high/medium and 1 = low).

Variables eventually associated with fitness level and single components addressed in the survey included initially 87 items. Since including too many variables in the model for LCA could negatively affect the analysis, a total of 18 variables (included in Table 1) out of the 87 items contained in the ASSO questionnaires were selected and gathered into the following three dimensions: (1) biological and genetic; (2) sociocultural and environmental; (3) life habits. The inclusion of items in the different dimensions was based on previous literature suggesting risks within the context of a larger conceptual framework for health characteristics and for sedentary behaviours [50,51,52] and subsequently was adapted to fitness.

Moreover, among these factors, a total of eleven latent class indicators were chosen to represent multiple dimensions of fitness risks, i.e., biological (health risk/status) and lifestyle (physical activity/sedentariness, alcohol/smoking, and meal patterns and habits) dimensions; the other seven variables were investigated in a multivariate analysis for their role as possible predictors of class membership (gender, age, having at least one parent overweight/obese, parents’ education, family affluence scale (FAS), town of residence’s size, and school type) [50,51,52,53].

Binary latent class indicators were created for convenience of use, with a recoding scheme that dichotomized fitness-related behaviours, with ‘0’ representing a healthier behaviour and ‘1’ representing a less healthy behaviour.

Among the biological and genetic determinants, weight status, health risk, malaise frequency and diagnosed diseases were selected for the latent classes’ assessment, while sex, age, and having at least one parent overweight/obese were selected as predictors.

### 2.3. LCA and Logistic Regression Analyses

An LCA was performed to identify latent classes of fitness-related risk behaviours. The number of classes that best fit the data was chosen by evaluating an increasing number of classes, through the log likelihood, Akaike information criterion (AIC), Bayesian information criterion (BIC) [23], sample-size-adjusted Bayesian information criterion (adjusted BIC), and a consistent version of AIC (CAIC) [54]. Latent classes were then defined by the probabilities that individuals in each class met the criteria for the considered eleven variables.

The chi-squared test was applied to assess differences between the classes, and multiple logistic regression models were used to examine the relationship between latent classes and predictors and between latent classes and overall fitness level and single fitness components. All regressions were controlled for potential confounders, and adjusted odds ratios (Adj ORs) were obtained.

Alpha level was set at 0.05, and 95% CIs were calculated for the ORs derived.

In this study, Stata/MP 12.0 software was used, and LCA was performed using the LCA Stata Plugin (PennState).

The ethical approval was given by the ethical committee of the “Azienda Ospedaliera Universitaria Policlinico Paolo Giaccone” (approval code n.9/2011). All the participant students provided an informed consent form signed by their parents.

## 3. Results

### 3.1. Sample Characteristics

A statistical description of the possible determinants of fitness levels across the population sample is provided in Table 1.

The fitness level and fitness component details in the whole sample and by gender and age are provided in Table 2, and indicate that males perform significantly better than females, and they also show an incremental trend amongst fitness levels with age in all physical components. These correlations with continuous data of fitness components and further details are presented in a previously published paper [45].

The fit statistics for two- to six-class LCA models are shown in the Supplementary Table S1. The log likelihood values and the information criteria index AIC decreased substantially for models with two to five classes and moderately for additional classes. The adjusted BIC of the five-class solution had the lowest values and subsequent class addition showed an increase in its values. Thus, the five-class model was selected as the best model and used in subsequent analyses.

The following five classes were identified: class 1—virtuous; class 2—low PA/sport; class 3—incorrect alcohol/food habits; class 4—health risk/overweight; class 5—malaise/diseases.

Table 3 shows the conditional probabilities of unhealthy lifestyles and health risks/unhealthy status across different classes of fitness-related risk behaviours.

The first latent class (“Virtuous”) represents 30.7% of respondents. Almost all adolescents in this class show good fitness levels and physically active behaviours, with a high percentage practicing sport for 3 or more h/week. They have the lowest probability of screen-watching for more than 2 h/day and of having inadequate meals and food habits. None of them are at health risk and almost all of them have low malaise frequency and do not have diagnosed diseases. Almost 90% show a correct weight status.

The second latent class (“Low PA/sport”, 18.8% of respondents) is characterized by low levels of PA and all adolescents belonging to this class practice sport less than 3 h/week. The majority (60.8%) have nonadequate meals and around 31% are overweight/obese.

The third class (“Incorrect alcohol/foods habits”) is represented by 25.8% of respondents, with 77% of adolescents usually drinking alcohol at least 3 times/week, 94% having nonadequate meals, and 76% nonadequate food habits.

With regard to adolescents belonging to the fourth class (“Health risk/overweight”, 15.9%), 80% have higher risk of overweight/obesity and almost all of them are at health risk (99.9%). A total of 66% drink usually alcohol at least 3 times/week.

The last class (“Malaise/diseases”, 8.8%) is made of subjects reporting the highest probability of excessive malaise frequency, and around 70% of them have diseases diagnosed by a medical doctor. Around 40% of them are overweight/obese, 32.6% at health risk, and 66% usually drink alcoholics. With the exception of the first virtuous class, half of the sample population in each class watches TV, plays PC or videogames more than 2 h/day. Smoking is also equally distributed among classes, with 10% to 18% of subjects being current smokers.

### 3.2. Predictors of Latent Class Membership

Table 3 shows the frequencies of predictors for each class; the relative associations of the five identified latent classes with predictors are showed in Table 4.

Subjects from class 2 compared to class 1 are mainly older, come from low educated families, and attend professional/technical schools (Table 4).

Classes 3 and 5 present a higher probability of having male adolescents compared to class 1 (Table 4). Class 3 also has high odds of having older adolescents, with OR 2.53, 95% CI 1.71–3.75, *p* < 0.001. In class 5, adolescents are more likely to have at least one parent overweight/obese, low educated parents, and attend mostly technical/professional schools compared to class 1 (Table 4).

### 3.3. Fitness Level and Fitness Components by Latent Classes

In Table 4, the associations between latent classes and fitness levels and the single five fitness components assessed through the ASSO-FTB are shown, with ORs adjusted for gender, age, education, having at least one parents overweight/obese, and school type.

The poorest fitness levels were found in adolescents belonging to class 2—low PA/sport (20.0%). In the logistic regression with class 1 being the reference, subjects with low PA and sport practising from class 2 have ten times higher significantly likelihood of having poor fitness levels (Adj OR 10.39, 95% CI 4.46–24.21, *p* < 0.001) (Table 4).

Despite their high usual alcohol consumption and inadequate food habits, a total of 21.8% of adolescents from class 3 have high fitness levels, but a nonsignificant difference was found for fitness levels when compared to class 1 (Table 4).

Despite classes 4 and 5 showing, respectively, 19.0% and 26% of subjects with high fitness levels and 12.0% and 7.4% with low fitness levels, poor fitness levels were found to be significantly higher than class 1, with Adj OR 10.31, 95% CI 3.52–30.19, *p* < 0.001 and Adj OR 2.57, 95% CI 1.03–6.42, *p* < 0.05, respectively (Table 4).

The descriptive analysis of fitness components by latent classes shows that higher frequencies of low abilities were found in classes 2 and 4, while classes 1 and 3 showed fewer subjects with low fitness levels.

When analysing associations with single fitness components, significant higher risk of having all low health-related fitness components, with the exception of the upper body maximal strength, were found for class 2—low PA/sport (Table 4).

Adolescents in class 4 had a higher risk of low muscular endurance (Adj OR 2.67, 95% CI 1.26–5.64, *p* < 0.05), while class 5 showed significantly increased risk of lower body maximal strength, muscular endurance, and speed and agility (Table 4).

## 4. Discussion

The present data describe underlying patterns of HRF factors and their sociodemographic and environmental predictors, and report on the association of latent classes with fitness level and single fitness components.

Cluster analysis has been previously adopted to identify potential patterns of health-related behaviours in adolescents [31,32,33,34,35], and LCA has also been frequently used to determine patterns of behaviours on different population targets [26,27,36,37,38,39,40,41]. To our knowledge, there are no studies investigating fitness patterns in adolescents. Therefore, although comparison of our results with findings from other studies is limited, this study adds important information on possible predictors of low fitness levels in adolescents.

Five latent classes of fitness-related lifestyle patterns and health risk/status have been identified in our model. A first class was composed mainly of virtuous subjects, and this is consistent with one study on patterns of PA, sedentariness, and diet that identified a “healthful” class characterized by participants meeting recommendations for PA, consuming healthy foods, and showing a high overall health status and life satisfaction or low depression [22,26]. Moreover, other studies found a positive association of high PA levels, low sedentariness, and healthful diet with other physical and psychological factors in children, adolescents, and adults [30,55]. With regard to latent class 2, the major risk for unhealthy behaviours in this class was in the dimension of PA/sedentariness. The suboptimal food intake and overweight/obese status could be presumably related to their low PA and sport practice. Of note, subjects from this class were mostly males, older (more than 16 years), had low educated parents, and attended professional/technical vocational schools. This is consistent with findings from other studies that found that younger participants were generally more active than older youth [56,57] or that adolescents with low socioeconomic status (SES) were less active [58,59]. These observations suggest that interventions to enhance PA and sport involvement, which in turn may improve food intake and weight status, could be addressed to help fighting the global pandemic of physical inactivity among children and adolescents [60].

Moreover, it is likely that the lack of daily PA in this class could contribute to lower fitness levels and lower fitness abilities (excepted for upper body maximal strength) assessed through the ASSO-FTB reported in this class. This is also consistent with latent class 5, where almost all adolescents comply with the PA recommendations and a small number had low fitness levels. These results are in line with several studies. Morrow et al. in 2013 showed that adolescents failing to meet national aerobic and muscle-strengthening PA guidelines have higher odds of not achieving healthy physical fıtness levels of aerobic capacity [2]. Other longitudinal studies showed that PA interventions and PA combined with nutrition interventions were effective at increasing fitness levels in school-age youth [61,62,63]. Of interest, Silva et al. recently reported an association between hand-grip strength, body mass, body height, and PA levels in youth. The authors found that performance on these measures was positively related with greater body mass (probably muscle mass) and greater height (probably reflecting greater leverage) and only partially related with PA levels [64]. Collectively, these findings can indirectly explain the lack of association found in our study between latent class 2 and low upper body maximal strength (Table 4).

In the third latent class, unhealthy lifestyles characterize a high proportion of adolescents, who are mostly males and older compared to latent class 1, and this could explain the higher frequency of alcohol consumption that is generally more common in males and older adolescents [65] and poor food habits that are more common in males [66]. Despite these unhealthy behaviours, there is a high proportion of adolescents belonging to this class that have a high fitness level. This pattern was also found in one study [26], where adolescents from an “unhealthy” class were found to consume huge quantities of energy-dense foods, while not taking advantage of the health benefits associated with their more active lifestyle [39,55,67,68]. Moreover, since no association was found for fitness level and for all single fitness components in this class compared to the virtuous class, it is possible that these behaviours are not mere determinants of fitness levels. Currently, there is not a consensus on the relationship between fitness/PA and alcohol consumption in adolescents [65,69,70]. One study showed that adolescents with low upper-body musculoskeletal strength had a lower risk of alcohol consumption [71].

Thus, this alcohol-related behaviour should be further analysed before suggesting strategies and interventions aimed at decreasing the risk of low fitness. When, for example, latent class 4 is considered, where almost all subjects are at health risk, a strong association was found with fitness level; health risk was assessed through two components, one of this being the alcoholic risk; it could be suggested that in itself, drinking more than 12 g of ethanol per day could be a risk factor for low overall fitness level, while drinking alcoholic beverages in amounts <12 g of ethanol per day is not a risk factor in people more than 15 years old [72]. Moreover, adolescents from latent class 4 are more likely to be overweight/obese. The findings of the strong positive association evidenced between this class and low fitness level are in line with other studies confirming that fatness is inversely related to fitness [18,73,74]. A positive association in latent class 4 was also found with low muscular endurance. In accordance with Chen et al., our study confirms that overweight/obese adolescents have poorer performance in muscle endurance tests [75].

Subjects from latent class 5 are characterized by a health status that can be interrelated to the high probabilities of being overweight/obese, being at health risk, and practising sport less than 3 h/week that could be found in this class, as well as drinking alcohol. For example, high rates of adolescent sedentary behaviours have been associated with patterns of physical and psychological health in one study by Ussher et al. [76]. Different variables were found as predictors of this class, and this is in line with several studies that found children and adolescents with highly-educated parents as being more likely to display positive psychological health and fewer health complaints than youth with less educated parents, which highlights the need for programs helping people access university studies [77]. Although adolescents from class 5 show the highest percentage of high fitness levels, they are significantly more at risk of lower body maximal strength, muscular endurance, and speed and agility compared with class 1. This is partially in line with findings from other authors showing that children and adolescents with both upper- and lower- body muscular fitness had higher ORs of reporting fair (vs. excellent) perceived health status [78,79].

Importantly, it should be noted that the relationship between malaise and poor fitness level could be reciprocal. Generally, it could be hypothesised that the relationship is indirect, with those with a high malaise level, frequent health complaints, and diseases diagnosed by a medical doctor, and at the same time drinking alcohol and tending to be overweight, would likely have a less active lifestyle, which could lead to a low overall fitness level. Of interest, Farooq et al. recently reported that moderate-to-vigorous physical activity (MVPA) begins to decline at age 7 years [80], so it may be too late to start interventions during adolescence. This is an important public health message as interventions need to start early in life before risk factors such as low PA/sport (class 2) or incorrect alcohol/food habits (class 3) become present. Moreover, our research findings support the modern-day concept of exercise deficit disorder, which is aimed at identifying children with low levels of MVPA before they become more resistant to exercise interventions during adolescence [14,81].

It can be suggested that subgroups of adolescents with high malaise frequency and diseases, coming from low educated families, and attending technical/professional schools could be targets of interventions aimed at improving fitness levels and, in particular, muscular fitness. Secular trends indicate levels of muscular fitness in contemporary youth are decreasing [82], and therefore targeted interventions are needed to address growing concerns related to paediatric dynapenia in youth [21]. In our study, there was not a particular class characterized by a high level of sedentary behaviours, and this is in contrast with other studies comparing PA, sedentary behaviour, and diet contributing to obesity. One study analysing PA and sedentary behaviours [38] found three distinctive classes for boys and girls of active, sedentary, and low/moderate PA behaviour; another study applied LCA to PA and sedentary behaviours, showing a model with five patterns for average intensity, sedentary behaviour, light activity, MVPA, and vigorous activity [37]. However, since time spent watching TV, PC, and videogames are equally distributed in almost all classes of our study (from classes 2 to 5), interventions to increase fitness could convey the message of reducing screen time to all the subgroups identified. 

One of the main strengths of the present study is that it used the LCA to identify latent patterns underlying possible fitness-related behaviours, which has been demonstrated to be a valid approach to clustering subjects with similar characteristics. Risk factor clustering is an important tool in terms of public health and prevention to plan targeted interventions early in life. The choice of the LCA as the method of analysis was guided by the numerous categorical variables originally collected within the project through the ASSO toolkit. Compared to other clustering methods, this model-based clustering approach allows deriving clusters using a probabilistic model that describes distribution of data through a top-down approach. The classes’ identification can effectively improve our understanding of specific joint behaviours that should be modified to improve the health of school-age youth [27]. 

Moreover, the logistic regression analysis for the association of the latent patterns with overall fitness level and single fitness components was carried out for the purpose of checking the validity of the identified latent classes, and this is a valid approach for addressing proper strategies and interventions. Another strength of this paper is that the different fitness components were assessed through the ASSO-FTB, a validated tool that assessed various health-related fitness components [46]; these components were subsequently used to evaluate the overall fitness level through a principal components analysis (PCA) described in detail in another previous published paper [45]. One of the limitations of the present study is that for the assessment of PA and sedentariness, a validated web-based questionnaire was used with self-reported information, but no motor skills, muscular strength, or MVPA levels have been directly assessed. Moreover, statistical testing power of the regression analyses could have been decreased for some predictors, such as non-sedentary activities, FAS, or town size, because one of the groups of the binary variables was too small. It has to be also considered that dichotomizing variables, even if this is a common approach applied in LCA, could have reduced sensitivity and lost some key information. Another limitation is that the study sample was composed of adolescents from an area in Southern Italy, thus the sample was not representative of the entire national population. This did not allow the comparison of behaviours with adolescents from the northern and central parts, and can help with suggesting strategies that are valid at the local but not at the national level.

## 5. Conclusions

Data from this study can be used to help designing and implementing multifactorial interventions aimed at enhancing fitness levels and fitness abilities in school adolescents and consequently contribute to the prevention of chronic diseases in adulthood.

Both parental education and structured fitness interventions that enhance fundamental movement skills and muscular fitness in the school environment (e.g., physical education) as well as support for community-based outdoor active play and sport activities could be adopted. The risk of low physical fitness levels in adolescents could be reduced by daily participation in MVPA, regular physical education and sport practices, improvement of weight status and control of abdominal obesity (i.e., waist circumference), discouragement from using alcohol, education on correct food behaviours and habits, and improving understanding of their psychophysical malaise symptoms. Future studies within the ASSO project can be used to obtain representative national data in order to support the planning of appropriate strategies to enhance health behaviours and improve fitness abilities in youth.

## Figures and Tables

**Table 1:** Descriptive statistics of possible fitness level determinants in adolescents from the ASSO project (N = 883).

**Biological/Genetic**					
	**N**	**%**		**N**	**%**
**Gender**			**Age**		
Male	550	62.3	<16 years	331	37.5
Female	333	37.7	≥16 years	552	62.5
**Weight status**			**Overweight/obese parents**		
Normal/under	583	71.9	None	227	31.0
Overweight/obese	228	28.1	At least one	506	69.0
**Malaise frequency**			**Diagnosed diseases**		
Never/rarely	589	83.1	No	651	87.1
Weekly/daily	120	16.9	Yes	96	12.9
**Health risk** ^a^					
No	629	73.7			
Yes	225	26.3			
**Sociocultural and environmental**					
	**N**	**%**		**N**	**%**
**Parents’ education** ^b^			**Family affluence scale**		
High school or higher	443	60.3	Medium/high	718	96.1
Middle school or lower	292	39.7	Low	29	3.9
**Town of residence’s size**			**Study course type**		
Big centre	672	90.0	Lyceum	402	45.5
Small centre	75	10.0	Professional/technical	481	54.5
**Life habits**					
*Physical activity/Sedentariness*					
**Non sedentary activities**^c^ (min/day)			**Sport** (h/week)		
60 or more	726	93.4	3 or more	477	61.8
Less than 60	51	6.6	Less than 3	295	38.2
**Total screen time (h/day)**					
Less than 2	448	57.1			
2 or more	336	42.9			
*Alcohol consumption/Smoking*					
**Drinking alcoholics**			**Smoking**		
No	276	35.8	Never smoked/former smoker	665	86.1
Yes	496	64.2	Current smoker	107	13.9
*Meal patterns/Habits*					
**Meals’ adequacy** ^d^			**Food habits** ^e^		
Yes	336	43.5	Correct	388	50.3
No	436	56.5	Incorrect	384	49.7

^a^ Adolescents at health risk are those who are both at metabolic risk (estimated through the waist circumference percentiles) and alcoholic risk (i.e., drinking 12 g or more of ethanol per day). Health risk is referred to the possibility of developing chronic diseases, such as cardiovascular, alcohol-related, and other metabolic diseases. ^b^ Parents’ middle school or lower education was defined by the presence of at least one parent with middle school or lower education. ^c^ Non sedentary activities: getting ready in the morning, walking or biking to school or back from school, practising physical activity at school, doing home-based physical jobs (cleaning, hovering, cooking, etc.), outdoor activities or walking during free time, and practising sport. ^d^ Meals’ adequacy consisted of the presence of at least 3 adequate main daily meals. A proper meal excluded carbonated and sugar-sweetened drinks or “junk food” for breakfast or morning and afternoon breaks; and a proper meal including a first or a second course with vegetables, fruit, bread, and excluding carbonated and sugar-sweetened drinks or junk food, for lunch and dinner. ^e^ Food habits were derived by summing up eating out, eating ready meals, eating organic food, eating fresh food, and eating food from vending machines. They were considered “correct” if subjects had at least 4 correct behaviours among those.

**Table 2:** Fitness levels and health-related fitness components in the total sample and by gender and age.

	Total		Gender				Age			
			Males		Females		Age < 16		Age ≥ 16	
**Fitness level ^a^**	N	%	N	%	N	%	N	%	N	%
Low	77	14.2	25	6.8	52	29.7	33	16.8	44	12.7
Medium	369	67.8	257	69.6	112	64.0	134	68.0	235	67.7
High	98	18.0	87	23.6	11	6.3	30	15.2	68	19.6
	544		369		175		197		347	
**Upper body maximal strength ^a^**										
Low	125	16.1	76	15.8	49	16.5	49	17.2	76	15.4
Medium	504	64.8	299	62.0	205	69.3	180	63.2	324	65.7
High	149	19.1	107	22.2	42	14.2	56	19.6	93	18.9
	778		482		296		285		493	
**Lower body maximal strength**										
Low	141	18.6	92	19.6	49	17.1	61	21.8	80	16.8
Medium	436	57.7	264	56.3	172	59.9	152	54.5	284	59.5
High	179	23.7	113	24.1	66	23.0	66	23.7	113	23.7
	756		469		287		279		477	
**Muscular endurance**										
Low	110	15.8	72	16.7	38	14.3	42	16.9	68	15.2
Medium	453	65.1	270	62.6	183	69.1	166	66.7	287	64.2
High	133	19.1	89	20.6	44	16.6	41	16.4	92	20.6
	696		431		265		249		447	
**Speed and agility**										
Low	137	19.6	82	18.8	55	21.1	51	20.1	86	19.4
Medium	429	61.5	263	60.2	166	63.6	159	62.6	270	60.8
High	132	18.9	92	21.1	40	15.3	44	17.3	88	19.8
	698		437		261		254		444	
**Aerobic capacity**										
Low	136	22.0	91	22.4	45	21.1	52	23.9	84	21.0
Medium	333	53.8	216	53.2	117	54.9	109	50.0	224	55.9
High	150	24.2	99	21.4	51	23.9	57	26.1	93	23.1
	619		406		213		218		401	

^a^ significant difference within gender.

**Table 3:** Conditional probabilities and descriptive statistics of unhealthy lifestyles and health risks/unhealthy status across classes of fitness-related risk behaviors.

	Class 1: Virtuous (30.7%)		Class 2: Low PA/Sport (18.8%)		Class 3: Incorrect Alcohol/Food Habits (25.8%)		Class 4: Health Risk/Overweight (15.9%)		Class 5: Malaise/Diseases (8.8%)	
**PA/Sedentariness**										
Moderate PA < 60 min/day	0		0.335		0.016		0		0.004	
Sport < 3 h/week	0.179		1		0.282		0.257		0.367	
Screen watching > 2 h/day	0.275		0.462		0.507		0.498		0.518	
**Alcohol/Smoking/Food habits**										
Alcohol consumption	0.570		0.556		0.769		0.655		0.662	
Smoking	0.183		0.095		0.135		0.098		0.165	
Nonadequate meals	0.323		0.608		0.939		0.430		0.458	
Nonadequate food habits	0.313		0.455		0.756		0.488		0.457	
**Health risk/Status**										
Overweight/obese	0.132		0.307		0.049		0.800		0.389	
At health risk	0		0.215		0.137		0.999		0.326	
High malaise frequency	0.041		0.197		0.131		0		0.999	
Diagnosed diseases	0.067		0.035		0.085		0.117		0.691	
**Predictor frequencies**										
	N.	%	N.	%	N.	%	N.	%	N.	%
Males *	184	58.4	132	63.2	95	68.3	71	57.3	31	79.5
Age ≥ 16 **	183	58.5	135	64.6	92	66.2	73	59.8	30	76.9
At least one parent ow/ob	160	67.5	127	70.6	86	74.1	66	67.3	26	74.3
Low parents’ education	94	39.5	66	36.7	53	45.7	41	41.4	16	45.7
Low FAS	14	5.8	5	2.7	3	2.5	3	3.0	0	0.0
Technical/professional school *	170	54.0	110	52.6	90	64.7	69	55.6	21	53.8
Small town of residence	27	11.2	23	12.6	8	6.7	10	10.0	5	14.3

Note: (*) indicate significant differences between classes; percentage frequencies are calculated on the total subjects within each class. * *p* < 0.05; ** *p* < 0.01; PA: physical activity; ow/ob: overweight/obese; FAS: family affluence scale.

**Table 4:** Associations between latent classes and possible predictors of class membership.

	Class 1: Virtuous (30.7%)	Class 2: Low Pa/Sport (18.8%)	Class 3: Incorrect Alcohol/Food Habits (25.8%)	Class 4: Health Risk/Overweight (15.9%)	Class 5: Malaise/Diseases (8.8%)
**Predictors**		OR (95% CI)	OR (95% CI)	OR (95% CI)	OR (95% CI)
Gender (males vs. females)	ref.	0.79 (0.55–1.17)	1.95 (1.33–2.87) **	NS	2.88 (1.84–4.52) ***
Age (≥16 vs. <16)	ref.	1.80 (1.20–2.69) **	2.53 (1.71–3.75) ***	NS	1.20 (0.81–1.77)
At least one parent ow/ob ^a^ (yes vs. no)	ref.	NS	NS	NS	1.74 (1.08–2.79) *
Parents’ education (low vs. high)	ref.	1.94 (1.26–2.98) **	NS	NS	1.64 (1.07–2.52) *
FAS ^b^ (low vs. high)	ref.	NS	NS	NS	NS
Town size (small vs. big)	ref.	NS	NS	NS	NS
School type (technical/professional vs. lyceum)	ref.	1.75 (1.19–2.59) **	NS	NS	1.91 (1.28–2.84) **
**Fitness level (low vs. medium/high)**	ref.	10.39 (4.46–24.21) ***	NS	10.31 (3.52–30.19) ***	2.57 (1.03–6.42) *
**Fitness components**					
Upper body maximal strength ^c^	ref.	NS	NS	NS	NS
Lower body maximal strength ^d^	ref.	4.24 (2.54–7.08) ***	NS	NS	3.22 (2.00–5.18) ***
Muscular endurance ^e^	ref.	3.28 (1.93–5.58) ***	NS	2.67 (1.26–5.64) *	2.54 (1.57–4.11) ***
Speed and agility ^f^	ref.	2.25 (1.33–3.83) **	NS	NS	1.91 (1.66–3.12) *
Endurance/Aerobic capacity ^g^	ref.	2.14 (1.21–3.76) **	NS	NS	NS

^a^ ow/ob: overweight/obese; ^b^ FAS: family affluence scale; ^c^ assessed through the hand-grip test; ^d^ assessed through the standing broad jump test; ^e^ assessed through the sit-up test to exhaustion; ^f^ assessed through the 4 × 10 m shuttle run test; ^g^ assessed through the 20 m shuttle run test; * *p* < 0.05; ** *p* < 0.01; *** *p* < 0.001; OR: odds ratio, adjusted for gender, age, education, having at least one parent overweight/obese, school type; NS: not significant.

## Supplementary Materials

The following are available online at http://www.mdpi.com/1660-4601/15/9/1933/s1, Table S1: Model fit statistics for the 2- to 6-class LCA models of fitness-related behaviours (N = 883).
