# Supplementary material for: Profiles of Physical Fitness Risk Behaviours in School Adolescents from the ASSO Project: A Latent Class Analysis
Source: Int J Environ Res Public Health. 2018 Sep 5;15(9):1933. doi: 10.3390/ijerph15091933 (PMC6163564; doi:10.3390/ijerph15091933)
Supplement: Supplementary file 1 [file ijerph-15-01933-s001.pdf]

Supplementary Table S1. Model fit statistics for the 2- to 6-class LCA models of fitness-related behaviours (N = 883).

	2 classes	3 classes	4 classes	5 classes	6 classes
Log likelihood	−4818,2413	−4783,3701	−4739,7451	−4698,5971	−4680,8938
G-squared	1205,4180	1135,6757	1048,4256	966,1297	930,7231
AIC[a]	1255,4180	1211,6757	1150,4256	1094,1297	1084,7231
BIC[b]	1375,7816	1394,6283	1395,9673	1402,2605	1455,4429
CAIC[c]	1400,7816	1432,6283	1446,9673	1466,2605	1532,4429
Adjusted BIC	1296,3851	1273,9456	1233,9984	1199,0054	1210,9016

[a] AIC = Akaike's Information Criteria; [b] BIC = Bayesian Information Criteria; [c] CAIC = consistent version of AIC.
